# Factors Associated With Knowledge and Experience of Self-managed Abortion Among Patients Seeking Care at 49 US Abortion Clinics

**DOI:** 10.1001/jamanetworkopen.2023.8701

**Published:** 2023-04-18

**Authors:** Abigail R. A. Aiken, Luisa Alejandra Tello-Pérez, Melissa Madera, Jennifer E. Starling, Dana M. Johnson, Kathleen Broussard, Elisa Padron, Carol Armelle Ze-Noah, Aleta Baldwin, James G. Scott

**Affiliations:** 1 Lyndon B. Johnson School of Public Affairs, University of Texas at Austin, Austin; 2 Bixby Center for Global Reproductive Health, Department of Obstetrics, Gynecology and Reproductive Sciences, School of Medicine, University of California, San Francisco; 3 Mathematica Policy Research Inc, Cambridge, Massachusetts; 4 Department of Sociology, University of South Carolina, Columbia; 5 Stanford School of Medicine, Stanford University, Palo Alto, California; 6 Charles and Louise Travers Department of Political Science, University of California Berkeley, Berkeley; 7 Department of Public Health, California State University Sacramento, Sacramento; 8 Department of Statistics and Data Sciences, University of Texas at Austin, Austin; 9 McCombs School of Business, University of Texas at Austin, Austin

## Abstract

**Importance:**

Patients attending US abortion clinics may consider or try self-managing their abortion before coming to the clinic, yet little is known about the factors associated with self-management behavior.

**Objective:**

To examine the prevalence and factors associated with considering or attempting a self-managed abortion before attending a clinic.

**Design, Setting, and Participants:**

This survey study included patients obtaining an abortion at 49 independent, Planned Parenthood, and academic-affiliated clinics chosen to maximize diversity in geographic, state policy, and demographic context in 29 states between December 2018 and May 2020. Data were analyzed from December 2020 to July 2021.

**Exposures:**

Obtaining an abortion at a clinic.

**Main Outcomes and Measures:**

Knowledge of medications used to self-manage an abortion, having considered medication self-management before attending the clinic, having considered any method of self-management before attending the clinic, and having tried any method of self-management before attending the clinic.

**Results:**

The study included 19 830 patients, of which 99.6.% (17 823 patients) identified as female; 60.9% (11 834 patients) were aged 20 to 29 years; 29.6% (5824 patients) identified as Black, 19.3% (3799 patients) as Hispanic, and 36.0% (7095 patients) as non-Hispanic White; 44.1% (8252 patients) received social services; and 78.3% (15 197 patients) were 10 weeks pregnant or less. Approximately 1 in 3 (34%) knew about self-managed medication abortion, and among this subsample of 6750 patients, 1 in 6 (1079 patients [16.1%]) had considered using medications to self-manage before attending the clinic. Among the full sample, 1 in 8 (11.7%) considered self-managing using any method before clinic attendance, and among this subsample of 2328 patients, almost 1 in 3 (670 patients [28.8%]) attempted to do so. Preference for at-home abortion care was associated with considering medication self-management (odds ratio [OR], 3.52; 95% CI, 2.94-4.21), considering any method of self-management (OR, 2.80; 95% CI, 2.50-3.13), and attempting any method of self-management (OR, 1.37; 95% CI, 1.10-1.69). Experiencing clinic access barriers was also associated with considering medication self-management (OR, 1.98; 95% CI, 1.69-2.32) and considering any method of self-management (OR, 2.09; 95% CI, 1.89-2.32).

**Conclusions and Relevance:**

In this survey study, considering self-managed abortion was common before accessing in-clinic care, particularly among those on the margins of access or with a preference for at-home care. These findings suggest a need for expanded access to telemedicine and other decentralized abortion care models.

## Key Points

**Question:**

How commonly do US patients seeking an abortion consider or attempt self-managed abortion before attending a clinic, and what factors are associated with self-management behavior?

**Findings:**

In this survey study of 19 830 patients attending 49 abortion clinics in 29 states, 1 in 8 considered self-managing before attending the clinic, and of these, 1 in 3 attempted to do so. Individual-level factors, including experiencing clinic access barriers and preferring at-home care, were associated with self-management behavior.

**Meaning:**

These findings suggest that considering self-managed abortion is a common part of the path to accessing care in a clinic, and those on the margins of clinic access or who prefer at-home care are more likely to self-manage.

## Teaser

This survey study evaluates the factors associated with knowledge and experience of self-managed abortion among patients seeking care at 49 US abortion clinics in 29 states.

## Introduction

Access to in-clinic abortion care in the US has changed profoundly following the *Dobbs v. Jackson Women’s Health Organization* Supreme Court decision, which overturned the constitutional right to choose abortion established in *Roe v. Wade*.^1^ In many states, access to clinical abortion care was already limited before the *Dobbs* decision. However, now state-level total or near-total bans have created large areas of the country where abortion in a clinic is unavailable.^2^ In this new policy climate, self-managed abortion has become an increasingly important component of access.^3^ A self-managed abortion is one that occurs outside the formal health care setting and comprises a spectrum of methods, including abortion medications (mifepristone and/or misoprostol), herbs and botanicals, and self-harm.^4^ Although people have self-managed their abortions throughout history, increased access to abortion medications via online pharmacies and telemedicine services such Aid Access means that more people may now self-manage using pills.^5^

Although self-managed abortion is often viewed solely as a mutually exclusive alternative to in-clinic care, research suggests that it may be considered or attempted by those who ultimately do access a clinic as part of the path to securing an accessible and acceptable option. A 2014 study showed that 1.2% of US abortion clinic patients had ever attempted to self-manage using misoprostol,^6^ while a 2019 study showed that abortion clinic patients in Texas considered or attempted self-management before attending the clinic because they were unsure whether they would be able to overcome access barriers or because they would have preferred to self-manage but were ultimately unable to find an acceptable method.^7^

Very little is known, however, about how considering or attempting self-managed abortion before accessing in-clinic care may vary by state and local context or by individual circumstances. To date, 12 states have implemented total or near-total abortion bans after *Dobbs*, and 12 more are expected to enact similar bans or other types of severe restrictions.^8^ With these new laws in place, knowledge of the factors that motivate self-management behavior among those attending clinics is important; groups who were already more likely to consider or attempt self-management before attending a clinic before *Dobbs* may also be more likely to self-manage when no clinics are available. Knowledge of these specific groups could allow public health practitioners to ensure that information on safe and effective self-management reaches vulnerable groups and could allow physicians in ERs, primary care settings, and OBGYN offices to be prepared to encounter more patients seeking advice or follow-up care for self-management. Using a survey of abortion patients from a sample of clinics across the US, the objectives of our study are to (1) assess knowledge of, interest in, and experiences with self-managed abortion among patients attending clinics in diverse policy, geographic, and sociodemographic contexts; and (2) examine the association of individual-level factors (such as sociodemographic characteristics, access barriers, and abortion care preferences) and clinic-level factors (such as geography and state policy climate) with self-management knowledge, interest, and experiences.

## Methods

Between January 2019 and May 2020, we conducted a survey of abortion patients in 46 independent, Planned Parenthood, and academic-affiliated clinics located in 28 states across the contiguous US. Three clinics located in Texas participated in a pilot study in 2018 to test the feasibility of data collection and are included for a final sample of 49 clinics in 29 states. The institutional review board at the University of Texas at Austin approved the study and participants provided anonymous informed consent. This study followed the American Association for Public Opinion Research (AAPOR) reporting guideline.^9^

We compiled a list of clinics cross-referenced from multiple sources and selected sites using a statistical algorithm designed to maximize sample diversity across 4 key clinic-level variables: (1) geographic region, defined according to the US Census categorization of regions and subregions^10^; (2) state abortion policy context, defined according to the 2018 Guttmacher Classification according to state legislation either restricting or supporting abortion access^11^; (3) distance from an international border (either Mexico or Canada), defined using the Google Maps Application Programming Interface; and (4) racial and ethnic diversity according to US Census data^12^ and later confirmed by selected clinics. Among our sample of 49 clinics, 12% were located in the Northeast, 22% in the Midwest, 29% in the West, and, 37% in the South. Fifty-five percent were in states with a hostile or extremely hostile abortion policy climate, while 45% were in states with a neutral or supportive policy climate. Forty-three percent of clinics were within 200 miles of either the Canadian or Mexican border, and 61% served primarily racial and ethnic minority groups. Participating clinics were offered $2000 in compensation for staff time.

We aimed to survey 500 patients per clinic, with the exception of 14 clinics with smaller than average monthly patient volume, where our goal was 250 patients. Calculations from our pilot study demonstrated that these sample sizes would provide us with 80% power to detect even fairly small differences in average outcomes between sites at an α level of .05. Our final target sample size was 20 850 patients.

All patients presenting for abortion care at each clinic were eligible to participate, except in states with parental consent laws for minors, where we implemented a minimum age requirement of 18 years. No compensation was offered for participation. Clinic staff informed patients about the survey and provided those interested with an iPad displaying information about the study as well as a consent form where patients provided anonymous informed consent. The survey was fielded using REDCap software and data were stored on a protected server. The survey was available in both English and Spanish and no identifying information was collected.

The survey included questions about patients’ knowledge of self-managed abortion, whether they had considered self-managing before coming to the clinic, and whether they had attempted to self-manage, both with respect to abortion pills and nonmedication methods of self-management. Further details on question sequence are shown in eFigure 1 in Supplement 1. Respondents who had knowledge of medication self-management were also asked about pill names and sources. Those who considered self-management were also asked about the reasons why they did so. All participants were asked about their experiences accessing abortion care in the clinic (including any barriers they encountered), their preferred model of abortion care (in-clinic vs models not involving an in-clinic visit, including telemedicine, pharmacy pick-up models, and self-management), previous abortion experiences, and whether they knew anyone who had ever self-managed using abortion medications. Finally, we asked participants a series of demographic questions, including age, duration of pregnancy, country of birth, number of children, education level, receipt of social services, gender identity, and racial and ethnic identity. Race and ethnicity were included because patients may have systematically different experiences accessing abortion due to structural racism. To represent racial and ethnic diversity while accounting for small sample sizes, we created 4 groups: Black, Hispanic, non-Hispanic White, and Other. The Other category includes respondents who identified as Asian, Multiracial, American Indian, or who checked the answer option “another identity” in the survey instrument.^13^ Detailed survey information can be found in eTable in Supplement 1.

### Statistical Analysis

We calculated the prevalence of knowledge, consideration, and use of medication self-management, as well as having considered or tried any method of self-management before coming to the clinic. We then conducted regression analyses to examine the individual and clinic-level factors associated with each of 4 binary outcomes of interest: (1) knowledge of medication self-management; (2) having considered medication self-management before visiting the clinic; (3) having considered any method of self-management before visiting the clinic; (4) having tried any method of self-management before visiting the clinic. We fit separate within-between logistic regression models^14^ to each of the 4 outcomes. Within-between models allowed us to account for the hierarchical nature of the survey data, where multiple respondents were surveyed within each participating clinic, and to eliminate bias resulting from between-clinic differences in respondent-level covariate associations. Although fixed-effects models with clinic dummy variables are also an option for dealing with such bias, these eliminate distinctive clinic characteristics, which are also of interest in our study.

For each regression model, we examined 3 categories of covariates. First, variables summarizing individual-level abortion perspectives and experiences, including whether respondents faced barriers to clinic access, knew somebody who self-managed using pills, preferred a nonclinic model of care, or had previously had an abortion. Second, individual-level demographic characteristics, including whether a respondent was born outside the US; had 1 or more children; identified as Black, Hispanic, non-Hispanic White, or another racial or ethnic group; and received any social services. Third, clinic-level characteristics, including proximity to an international border, serving a predominantly racial and ethnic minority population, and state policy context. We evaluated odds ratios (ORs) using α = .05 to assess statistical significance. We used R version 4.0.2 (R Project for Statistical Computing) to conduct all statistical analyses and the package panelr to fit our models. Data were analyzed from December 2020 to July 2021.

## Results

Overall, 19 830 patients participated in the survey, and 99.6.% (17 823 patients) identified as female, 60.9% (11 834 patients) were aged between 20 and 29 years, 43.6% (7796 patients) had attended some college, and 27.6% (4947 patients) had graduated high school only (Table 1). A total of 29.6% (5824 patients) identified as Black or African American, 19.3% (3799 patients) as Hispanic, 36.0% (7095 patients) as non-Hispanic White, and 15.2% as another group (other includes respondents who identified as Asian, Multiracial, American Indian, or who checked the answer option “another identity” in the survey instrument). Most respondents (11 701 [60.4%]) already had children, 8.6% (1665 patients) were born outside the US, and 44.1% (8252 patients) received social services. A total of 78.3% (15 197 patients) were 10 weeks pregnant or less. During recruitment, 26 531 patients were invited to participate in the survey and 19 830 agreed; 16 723 gave complete responses and 3107 partial responses, for a response rate of 75%. Our response rate counts partial survey completion as a response, aligned with AAPOR suggested practices.^9^ Missing data for each variable are listed in Table 1, Table 2, and Table 3.

**Table 1.** Demographic Characteristics of Patients Attending 49 Abortion Clinics in the US

Characteristic	Frequency (%) (N = 19 830)^a^
Age, y	
<20	1704 (8.8)
20-24	5766 (29.7)
25-29	6068 (31.2)
30-34	3674 (18.9)
35-39	1665 (8.6)
≥40	550 (2.8)
Country of birth	
US	17 645 (91.4)
Other	1665 (8.6)
Education	
Less than high school	229 (1.3)
Some high school	1312 (7.3)
Graduated high school	4947 (27.6)
Some college	7796 (43.6)
Bachelor’s degree	2820 (15.8)
Master’s degree or higher	797 (4.5)
Gender	
Female	17 823 (99.6)
Male	18 (0.1)
Trans, queer, nonbinary, or other	60 (0.3)
Gestation, wk	
≤10	15 197 (78.3)
11-16	2770 (14.3)
17-21	631 (3.3)
>21	84 (0.4)
Unsure	717 (3.7)
Parent	
Yes	11 701 (60.4)
No	7673 (39.6)
Race and ethnicity	
Black	5824 (29.6)
Hispanic	3799 (19.3)
White non-Hispanic	7095 (36.0)
Other^b^	2987 (15.2)
Receives social services	
Yes	8252 (44.1)
No	10 461 (55.9)

^a^ Missing data are as follows: age, 403 participants (2.0% of the sample); country of birth, 520 participants (2.6% of the sample); education, 1929 participants (9.7% of the sample); gender, 1929 participants (9.7% of the sample); gestation, 431 participants (2.2% of the sample); parent, 456 participants (2.3% of the sample); race and ethnicity, 125 participants (0.6% of the sample); receives social services, 1117 participants (5.6% of the sample).

^b^ Other includes respondents who identified as Asian (472 participants [2.4%]), Multiracial (1958 participants [9.9%]), American Indian (244 participants [1.2%]), or who checked the answer option “another identity” (313 participants [1.6%]) in the survey instrument.

**Table 2.** Individual-Level Abortion Perspectives and Experiences Among Patients in Our Sample Attending 49 Abortion Clinics in the US

Experience or perspective	Frequency (%) (N = 19 830)^a^
Clinic access barriers	
Yes	6237 (32.3)
No	13 060 (67.7)
Type of barrier^b^	
Abortion cost	3304 (53.0)
Taking time off work or school	2539 (40.7)
Distance to clinic	1760 (28.2)
Secrecy from family or partner	1503 (24.1)
Transportation	961 (15.4)
In-clinic appointment waiting time	751 (12.0)
Another barrier	349 (5.6)
Overnight stay	269 (4.3)
Prior abortion	
Yes	7192 (40.1)
No	10 721 (59.9)
Knows someone who self-managed using pills	
Yes	1515 (7.7)
No	16 183 (82.4)
Unsure	1946 (9.9)
Preferred abortion care model	
In-clinic	14 965 (81.6)
Not involving an in-clinic visit	3379 (18.4)

^a^ Missing data are as follows: clinic access barriers, 533 participants (2.7% of the sample); knows someone who self-managed an abortion using pills, 186 participants (0.9% of the sample); preferred abortion care model, 1486 participants (7.5% of the sample); prior abortion, 1917 participants (9.7% of the sample).

^b^ The number of patients experiencing barriers to access was 6237. Percentages do not sum to 100 as patients could choose more than 1 barrier.

**Table 3.** Knowledge and Experiences of Self-managed Abortion Among Patients in Our Sample Attending 49 Abortion Clinics in the US

Knowledge or experiences	Frequency (%) (N = 19 830)^a^
Knowledge of self-managed medication abortion	
Yes	6750 (34.1)
No	13 045 (65.9)
Name of medication known^b^	
Misoprostol	2912 (43.1)
Mifepristone	1613 (23.9)
Mifeprex	1075 (15.9)
Cytotec	864 (12.8)
Other	650 (9.6)
Cyrux	88 (1.3)
Star Pill	86 (1.3)
Artrotec	57 (0.8)
Mifegymiso	33 (0.5)
No name known	2420 (35.9)
Source of medication known^b^	
Online	1050 (15.6)
US pharmacy	769 (11.4)
Mexican pharmacy	347 (5.1)
Shop, mall or market	128 (1.9)
Local group	108 (1.6)
Friend or relative	89 (1.3)
Other pharmacy	53 (0.8)
Other	44 (0.7)
No known source	4433 (65.7)
Considered medication self-management^b^	
Yes	1079 (16.1)
No	5613 (83.9)
Considered any method of self-management	
Yes	2328 (11.7)
No	17 502 (88.3)
Methods considered^c^	
Medication abortion	1079 (46.3)
Vitamin C	807 (34.7)
Herbal remedies	715 (30.7)
Exercise	425 (18.3)
Alcohol	358 (15.4)
Injury	241 (10.4)
Other	115 (4.9)
Nonalcoholic beverages	77 (3.3)
Attempted any method of self-management^c^	
Yes	670 (28.8)
No	1658 (71.2)

^a^ Missing data are as follows: knowledge of self-managed medication abortion, 35 participants (0.2% of the sample); name of medication, 299 participants (4.4% of those with knowledge of self-managed medication abortion); source of medication, 72 participants (1.1% of those with knowledge of self-managed medication abortion); considered medication self-management, 58 participants (0.9% of those with knowledge of self-managed medication abortion).

^b^ Patients with knowledge of self-managed medication abortion totaled 6750. Percentages may not sum to 100 as patients could choose more than pill type and source.

^c^ Patients who considered any method of self-management totaled 2328. Percentages may not sum to 100 as patients could choose more than 1 method.

Individual-level abortion perspectives and experiences are shown in Table 2. Approximately one-third (6237 patients [32.3%]) had experienced barriers to clinic access; most commonly finding money to pay for care (3304 patients [53.0%]) and taking time off work or school (2539 patients [40.7%]). A total of 7192 participants (40.1%) had previously had an abortion and 1515 participants (7.7%) knew someone who had self-managed using pills. A total of 3379 participants (18.4%) had a preference for abortion care that does not involve in-person attendance at a clinic.

Among the full sample of 19 830 participants, 1 in 3 (6750 patients [34.1%]) knew about pills they could self-source and use to self-manage an abortion (Table 3). The most commonly known pill was misoprostol (2912 patients [43.1%]). Including other names for this medication—Cytotec, Artrotec, Cyrux, or Star Pill—increases this figure to 59.3% (4007 patients). Only 23.9% (1613 patients) knew of mifepristone. Most respondents who knew of pills lacked knowledge of any sources (4433 patients [65.7%]), but among those who did possess knowledge, online was the most common source (1050 patients [15.6%]).

Among the 6750 participants who knew about pills they could use to self-manage, 1 in 6 (1079 patients [16.1%]) had considered using them before attending the clinic (Table 3). The most common motivation was the cost of in-clinic care (518 patients [48.0%]), followed by a preference for the privacy of using pills at home (401 patients [37.2%]) (Figure).

**Figure.**
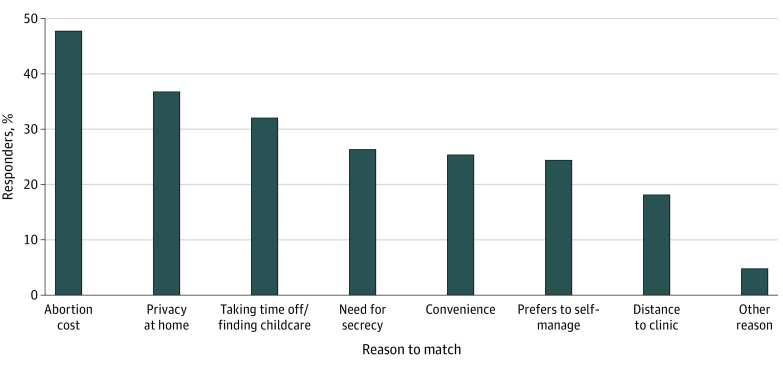
Reasons Why Respondents Considered Self-managed Medication Abortion Before Attending the Clinic Percentages do not sum to 100 because respondents could select more than 1 reason.

Among the full sample of 19 830 participants, 1 in 8 (2328 patients [11.7%]) considered self-managing using any method before attending the clinic. A total of 46.3% (1079 patients) considered using abortion pills, 34.7% (807 patients) considered vitamin C, and 30.7% (715 patients) considered herbs or botanicals (Table 3). Their motivations were similar to those who considered using pills only: cost (1429 patients [61.4%]) and preference for privacy (805 patients [35.0%]) (eFigure 2 in Supplement 1). Among the 2328 participants who considered self-managing using any method before attending the clinic, almost 1 in 3 (670 patients [28.8%]) attempted to do so (Table 3).

Regression models examining the associations of individual and clinic-level factors with knowledge of medication self-management (19 830 patients); consideration of medication self-management (6750 patients); consideration of any method of self-management (19 830 patients); and attempting any method of self-management (2328 patients) are shown in Table 4. We found the largest associations between individual-level abortion perceptions/experiences and self-management behavior. Experiencing clinic access barriers was associated with considering medication self-management (OR, 1.98; 95% CI, 1.69-2.32) and considering any method of self-management (OR, 2.09; 95% CI, 1.89-2.32) before attending the clinic. Knowing someone who had self-managed showed a similar pattern of associations with considering medication self-management (OR, 3.52; 95% CI, 2.93-4.23) and considering any method of self-management (OR, 4.40; 95% CI, 3.82-5.07). Preference for an at-home care model was associated with considering medication self-management (OR, 3.52; 95% CI, 2.94-4.21), considering any method of self-management (OR, 2.80; 95% CI, 2.50-3.13), and attempting any method of self-management (OR, 1.37; 95% CI, 1.10-1.69).

**Table 4.** Regression Models Examining Associations Between Individual and Clinic-Level Variables and Self-management Behaviors

Variable	Outcome	Odds ratio (95% CI)	*P* value
**Individual-level abortion perspectives and experience**			
Encountered barriers	Knowledge of medications for self-management^a^	1.11 (1.03-1.19)	.005
	Considered medication self-management^b^	1.98 (1.69-2.32)	<.001
	Considered any method of self-management^c^	2.09 (1.89-2.32)	<.001
	Tried any method of self-management^d^	1.14 (0.94-1.41)	.22
Knows somebody who self-managed using pills	Knowledge of medications for self-management	NA	NA
	Considered medication self-management	3.52 (2.93-4.23)	<.001
	Considered any method of self-management	4.40 (3.82-5.07)	<.001
	Tried any method of self-management	1.03 (0.79-1.35)	.70
Preferred care model does not involve clinic visit	Knowledge of medications for self-management	1.68 (1.62-1.75)	<.001
	Considered medication self-management	3.52 (2.94-4.21)	<.001
	Considered any method of self-management	2.80 (2.50-3.13)	<.001
	Tried any method of self-management	1.37 (1.10-1.69)	.004
Previous abortion experience	Knowledge of medications for self-management	1.10 (1.03-1.18)	.005
	Considered medication self-management	0.75 (0.63-0.88)	<.001
	Considered any method of self-management	0.96 (0.86-1.07)	.44
	Tried any method of self-management	1.45 (1.17-1.80)	<.001
**Individual-level demographics**			
Born outside US	Knowledge of medications for self-management	0.90 (0.79-1.02)	.10
	Considered medication self-management	0.83 (0.61-1.14)	.25
	Considered any method of self-management	0.94 (0.77-1.14)	.52
	Tried any method of self-management	0.99 (0.66-1.49)	.97
Parent	Knowledge of medications for self-management	1.01 (0.93-1.08)	.89
	Considered medication self-management	0.89 (0.75-1.06)	.19
	Considered any method of self-management	0.76 (0.68-0.85)	<.001
	Tried any method of self-management	0.65 (0.51-0.82)	<.001
Black race/ethnicity	Knowledge of medications for self-management	1.12 (1.03-1.22)	.009
	Considered medication self-management	0.93 (0.75-1.14)	.48
	Considered any method of self-management	1.11 (0.97-1.27)	.14
	Tried any method of self-management	0.81 (0.62-1.07)	.15
Hispanic race/ethnicity	Knowledge of medications for self-management	0.94 (0.83-1.06)	.32
	Considered medication self-management	1.14 (0.86-1.51)	.35
	Considered any method of self-management	1.03 (0.86-1.25)	.72
	Tried any method of self-management	1.62 (1.12-2.35)	.01
Other race/ethnicity	Knowledge of medications for self-management	1.08 (0.98-1.20)	.13
	Considered medication self-management	1.07 (0.85-1.36)	.55
	Considered any method of self-management	1.17 (1.00-1.36)	.06
	Tried any method of self-management	1.08 (0.80-1.47)	.61
Receives social services	Knowledge of medications for self-management	1.12 (1.04-1.20)	.002
	Considered medication self-management	1.00 (0.85-1.19)	.96
	Considered any method of self-management	1.21 (1.08-1.35)	<.001
	Tried any method of self-management	1.10 (0.88-1.38)	.41
**Clinic-level variables**			
Close to Mexican or Canadian border	Knowledge of medications for self-management	1.23 (1.05-1.44)	.009
	Considered medication self-management	1.08 (0.88-1.33)	.47
	Considered any method of self-management	1.04 (0.90-1.19)	.61
	Tried any method of self-management	0.63 (0.48-0.82)	<.001
Hostile abortion policy climate	Knowledge of medications for self-management	1.06 (0.89-1.27)	.52
	Considered medication self-management	0.95 (0.75-1.20)	.68
	Considered any method of self-management	0.98 (0.83-1.17)	.84
	Tried any method of self-management	0.76 (0.56-1.03)	.08
Serves majority minority population	Knowledge of medications for self-management	1.07 (0.90-1.28)	.42
	Considered medication self-management	1.08 (0.86-1.36)	.50
	Considered any method of self-management	1.30 (0.80-1.53)	.20
	Tried any method of self-management	1.00 (0.76-1.31)	.98

Abbreviation: NA, not applicable.

^a^ Patients who had knowledge of medications for self-management totaled 19 830.

^b^ Patients who considered medication self-management totaled 6750.

^c^ Patients who considered any method of self-management totaled 19 830.

^d^ Patients who tried any method of self-management totaled 2328.

Individual-level demographics showed some smaller associations with self-management behavior. Identifying as Black was associated with knowledge of medication self-management (OR, 1.12; 95% CI, 1.03-1.22), while identifying as Hispanic was associated with having tried any method of self-management (OR, 1.62; 95% CI, 1.12-2.35). Receiving any form of social services was associated with both knowledge of pills (OR, 1.12, 95% CI, 1.04-1.20) and having considered any method of self-management (OR, 1.21, 95% CI, 1.08- 1.38). Being a parent was negatively associated with having considered (OR, 0.76; 95% CI, 0.68-0.85) or tried any method of self-management (OR, 0.65; 95% CI, 0.51-0.82).

At the clinic level, location close to the Mexican or Canadian border showed a small positive association with knowledge of medication self-management (OR, 1.23; 95% CI, 1.05-1.44) but a negative association with having tried any method of self-management (OR, 0.63, 95% CI, 0.48-0.82). None of the other clinic-level characteristics were significantly associated with any of the 4 outcomes.

## Discussion

In this study of 19 830 patients seeking abortion care in 49 US clinics, we found that knowledge of medication self-management is prevalent and that considering self-managed abortion is a common part of the path to accessing in-clinic abortion care. Considering self-management is associated with both clinic access barriers and a preference for at-home abortion care, and these individual-level factors show larger associations with self-management behavior than clinic-level contextual factors or individual-level demographic factors.

The larger association of individual-level (as opposed to clinic-level) access barriers suggests that although abortion restrictions in some states no doubt make access challenging, the cost and logistics of in-clinic care can be obstacles for patients everywhere. Most people pay out-of-pocket for their abortions due to the Hyde Amendment,^15^ restrictions on public spending, and because private insurance rarely covers abortion.^16^ In 2020, the national median self-pay price for medication abortion was $560.^17^ Abortion clinics across the country continue to unify efforts to provide abortion care to nearby populations affected by abortion restrictions, and these efforts will become even more important as more people travel to access abortion care out-of-state.^18^ However, our findings suggest that many of those who considered self-managed abortion before ultimately attending a clinic did so because they were already on the margins of clinic access. It thus seems likely that greater costs and travel distances following the *Dobbs* decision will make self-managed abortion a more realistic option than out-of-state travel. Additionally, as self-management becomes more prevalent, the number of people who know someone who has self-managed will increase, perhaps influencing their own behavior, as our results suggest.

The prominent association of individual-level preferences for at-home abortion with self-management behavior suggests that service delivery models designed to allow patients to choose medication abortion without an ultrasound or in-person consultation fill an important gap in patient-centered care. No-touch telemedicine provision for medication abortion following the FDA’s decision to end the in-person dispensing requirement on mifepristone is likely already meeting some of this demand in the states where it is available.^19^ Additionally, the recent FDA decision to allow mifepristone to be dispensed by pharmacists following prescription by a physician may further increase remote care options.^20^ Future pharmacist-only provision models and over-the-counter status for abortion medications could further increase access and meet patient preferences.^21,22^ However, these models are unlikely to profoundly change access for people living in states with total or near-total abortion bans, further increasing the importance of self-management in these states. Although one-third of our sample knew that they could self-source abortion pills and use them to self-manage, specific knowledge about exactly which types pills they might use and where they might acquire them was relatively low. This finding suggests that access to accurate information about medication self-management and trustworthy sources of pills is now an important focus for public health, in line with insights from prior work examining the concerns and priorities of people in the US seeking abortion medications online.^23,24^ In addition, the associations we found between race and ethnicity, receipt of social services, and self-management behavior demonstrate a need to ensure equitable access to accurate, unbiased, and culturally competent information.

### Strengths and Limitations

The main limitation of our study is that data are self-reported and could be subject to patient discomfort or concerns about sharing self-managed abortion experiences. However, survey self-administration and anonymity likely reduced reporting bias. A key strength is our large sample, designed to explore diversity in individual and clinic context. Although results are not necessarily generalizable to the national population, our aim was to explore differences between clinics with different geographical and policy environments and between individuals with different personal experiences and circumstances.

## Conclusions

In this survey study of 19 830 patients attending 49 abortion clinics in the US, we found that knowledge of medication self-management is prevalent and that considering self-managed abortion is a common part of the path to accessing in-clinic abortion care. Our study mirrors the findings of prior work that has demonstrated significant access barriers to US abortion care as well as emerging insights on patients’ preferred care models.^25,26^ We extend this literature to show that rather than being solely a mutually exclusive alternative to in-clinic care, considering self-managed abortion is also a common part of the path to accessing care in the clinic setting. For those who consider or attempt self-management due to a preference for at-home abortion care, new service delivery models that recenter patient desires will help to fill the gap in states where they can be implemented. For those who consider or attempt self-management due to clinic access barriers, safe, effective, and supported options for self-care have become an even more important component of the US abortion care landscape now that *Roe* is overturned. Public health practitioners will need to consider how to design and disseminate accessible, quality information about safe and effective self-management, while clinicians will need to be ready to encounter those seeking advice about self-management and those seeking safe, medically appropriate, and nonjudgmental aftercare.

## Notes

Supplement 1. **eTable. **Survey Instrument Questions and Answer Options **eFigure 1. **Flow Chart Showing the Sequence and Denominators of Survey Questions **eFigure 2. **Reasons Why Respondents Considered All Methods of Self-Managed Abortion Before Attending the Clinic

Supplement 2.
Data Sharing Statement
