# Supplementary material for: Factors Associated With Knowledge and Experience of Self-managed Abortion Among Patients Seeking Care at 49 US Abortion Clinics
Source: JAMA Netw Open. 2023 Apr 18;6(4):e238701. doi: 10.1001/jamanetworkopen.2023.8701 (PMC10114063; doi:10.1001/jamanetworkopen.2023.8701)
Supplement: Supplement 1. — eTable. Survey Instrument Questions and Answer Options eFigure 1. Flow Chart Showing the Sequence and Denominators of Survey Questions eFigure 2. Reasons Why Respondents Considered All Methods of Self-Managed Abortion Before Attending the Clinic [file jamanetwopen-e238701-s001.pdf]

Supplemental Online Content

Aiken ARA, Tello-Pérez LA, Madera M, et al. Factors associated with knowledge and experience of self-managed abortion among patients seeking care at 49 US abortion clinics. *JAMA Netw Open*. 2023;6(4):e238701.
doi:10.1001/jamanetworkopen.2023.8701

eTable. Survey Instrument Questions and Answer Options
eFigure 1. Flow Chart Showing the Sequence and Denominators of Survey Questions
eFigure 2. Reasons Why Respondents Considered All Methods of Self-Managed Abortion Before Attending the Clinic

This supplemental material has been provided by the authors to give readers additional information about their work.

© 2023 Aiken ARA et al. *JAMA Network Open.*

eTable. Survey Instrument Questions and Answer Options

Survey Section		Question	Answer options
**Self-Managed Abortion Medication**	**Knowledge**	Have you ever heard of pills that you can use to have an abortion at home, WITHOUT ever going to a clinic or seeing a doctor?<br>NOTE: These pills are NOT the same as Plan B or Emergency Contraception	Yes, No
	**Pill types known of** (If yes to pills knowledge)	Please choose the name of the pill or pills you have heard about<br>Please check ALL the pills you know about	Cytotec, Misoprostol, Star Pill, Cyrux, Artrotec, Mifepristone, Mifeprex, Mifegymiso, Other, I have heard of a pill but don't know the name of it, I have never heard of any of these pills
	**Pills sources known of** (If yes to pills knowledge)	Do you know where you could get any of these pills?<br><br>Please check ALL the places you know about	Yes, from an online source; Yes, from a pharmacy in Mexico; Yes, from a shop or mall; Yes, from a pharmacy in the United States; Yes, from a pharmacy in Canada; Yes, from a market; Yes, from a friend or family member; Yes, from a local group; Yes, from another source (please specify); No, I don't know where I could get the pills
	**Consideration** (If yes to pill knowledge)	Before you came to the clinic today, did you consider buying pills to have an abortion or end your pregnancy at home, without coming to the clinic?	Yes, No

© 2023 Aiken ARA et al. *JAMA Network Open.*

	Survey Section	Question	Answer options
	**Reasons why** considered pills at home	Please tell us a little more about why you considered buying the pills and using them at home instead of going to a clinic.<br><br>Please check ALL that apply	It was hard to find money to pay for an abortion at a clinic; It was hard to get to a clinic because it's very far away; It was hard to take time away from work, school, and/or family to get to the clinic; I could not tell my family that I was going to have an abortion; I prefer the privacy of using pills at home; I wanted to take care of my abortion myself; It would be more convenient to buy the pills myself and have my abortion at home; other reason
	**Attempt** (If yes to pill consideration)	Did you use the pills?	Yes, No
**Self-Managed Abortion with non-pill methods**	**Consideration**	Before you came to the clinic today, did you consider bringing on your period or ending your pregnancy yourself using some other method BESIDES pills?	Yes, No
	What non-pill method(s) considered	Which method or methods did you consider using?<br><br>Please check ALL the methods you considered	Vitamin C (supplements or food/drink); Herbal remedies (like parsley, cohosh, or dong quai); Beverages (like Malta); Alcohol; Physical exercise; Physical injury; Another method (please specify)

© 2023 Aiken ARA et al. *JAMA Network Open.*

	Survey Section	Question	Answer options
	**Reasons why** considered non-pill method(s)	Please tell us a little more about why you considered using this method/these methods at home instead of going to a clinic.<br><br>Please check ALL that apply	It was hard to find money to pay for an abortion at a clinic; It was hard to get to a clinic because it's very far away; It was hard to take time away from work, school, and/or family to get to the clinic; I could not tell my family I was going to the clinic and it was hard to get away; I prefer the privacy of using this method at home; I wanted to take care of my abortion myself; It would be more convenient to do my abortion at home; other reason
	**Attempt** (If yes to non-pill method(s) consideration)	Did you try using this method/these methods?	Yes, No
**Abortion experiences and perspectives**	Knows someone who Self-Managed an abortion	Has anyone you know ever tried to have an abortion using pills they bought themselves and used at home?	Yes, No, Unsure
	Previous abortion experience	Have you ever previously gotten abortion care at a clinic?	Yes, No

© 2023 Aiken ARA et al. *JAMA Network Open.*

	Survey Section	Question	Answer options
	Barriers to access abortion care at the clinic	Did you experience any difficulties getting abortion care at this clinic?<br><br>Please check ALL that apply	Yes, it was hard to find money to pay for the abortion; Yes, it was hard to take time away from work and/or school or find child care; Yes, the clinic was very far away; Yes, I had to find transportation to the clinic; Yes, I had to stay overnight; Yes, it was hard because I couldn't tell my family/partner where I was going; Yes, I had to wait a long time before the clinic could see me; I experienced another problem not listed here; No, it wasn't hard for me to get to the clinic
	Preferred Abortion pathway	Based on your experiences, what would be your preferred way to get abortion care?	Go to a clinic to get a surgical abortion; Go to a clinic to get a medication abortion; Have an abortion at home without going to a clinic, by getting pills at a pharmacy and getting instructions from a doctor by video or phone; Have an abortion at home without going to a clinic by getting pills by mail and getting instructions through email or live chat; Have an abortion at home without going to a clinic by getting pills by mail and getting instructions by calling a clinic help line; I would prefer a different way
**Individual Demographic Characteristics**	Age	What age are you?	Under 15; 15-19; 20-24; 25-29; 30-34; 35-39; 40-44; 45-50; Over 50
	Gestational age	How many weeks pregnant are you?	10 weeks or less; 11-16 weeks; over 16 weeks; I'm not sure

© 2023 Aiken ARA et al. *JAMA Network Open.*

	Survey Section	Question	Answer options
	Country of birth	In which country were you born?	U.S.; Mexico; Other country (please specify)
	Sexuality	Which of the following terms best describes your sexual orientation?	Heterosexual or straight; Homosexual, gay or lesbian; Bisexual; I identify as something else not listed here
	Number of children	How many children do you have?	0; 1; 2; 3; 4; 5; 6; 7; 8 or more
	Education level	What is the highest level of school that you have completed?	Less than high school; Some high school; Graduated high school; Some college; Bachelor's degree; Master's degree or higher
	Receipt of social services	Does anybody in your household receive any of the following programs? Please check ALL that apply	Medicaid; TANF (Welfare); CEAP; SNAP (Food stamps); WIC; CHIP (Children's Health Insurance Program); SSI (Social Security Insurance); Other (please specify)
	Ethnicity	Are you of Hispanic or Latino origin?	Yes, No
	Race	With what racial group do you identify? Please check ALL that apply	Black or African American; White; Asian; American Indian or Alaska Native India; Other (please specify)

© 2023 Aiken ARA et al. *JAMA Network Open.*

eFigure 1. Flow Chart Showing the Sequence and Denominators of Survey Questions

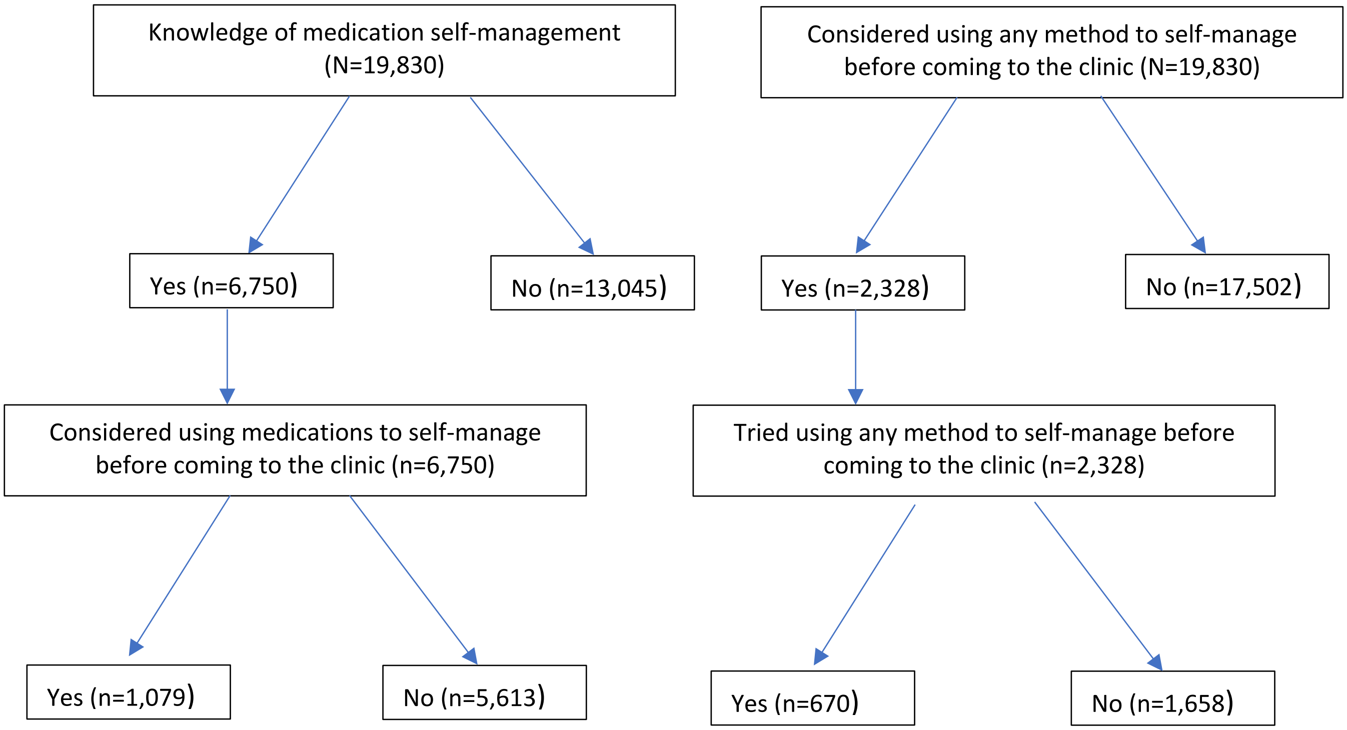

© 2023 Aiken ARA et al. *JAMA Network Open.*

eFigure 2. Reasons Why Respondents Considered All Methods of Self-Managed Abortion Before Attending the Clinic

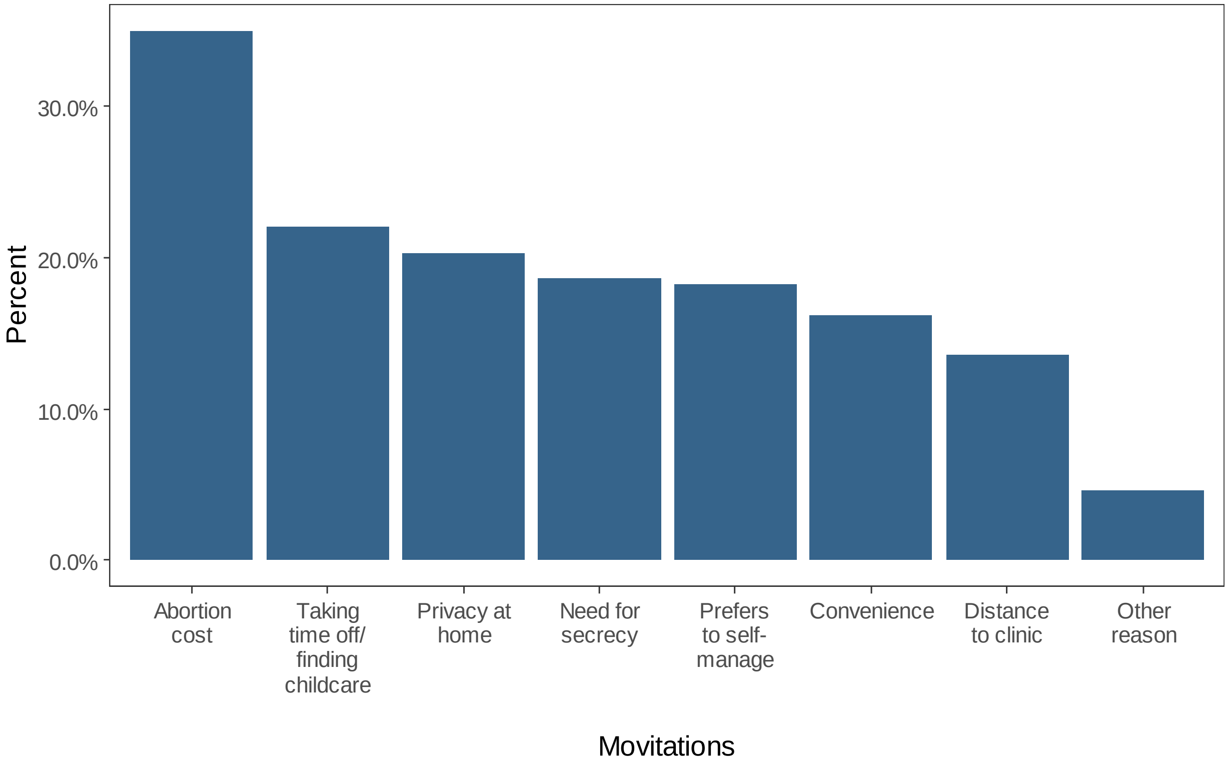

N=2,328. Percentages do not sum to 100 because respondents could select more than one reason

© 2023 Aiken ARA et al. *JAMA Network Open.*
