# Supplementary material for: Factors Associated With Knowledge and Experience of Self-managed Abortion Among Patients Seeking Care at 49 US Abortion Clinics
Source: JAMA Netw Open. 2023 Apr 18;6(4):e238701. doi: 10.1001/jamanetworkopen.2023.8701 (PMC10114063; doi:10.1001/jamanetworkopen.2023.8701)
Supplement: Supplement 2. — Data Sharing Statement [file jamanetwopen-e238701-s002.pdf]

Data Sharing Statement

Aiken. Factors Associated With Knowledge and Experience of Self-Managed Abortion Among Patients Seeking Care at 49 US Abortion Clinics. *JAMA Netw Open*. Published April 18, 2023. doi:10.1001/jamanetworkopen.2023.8701

Data
Data available: No
